# Changing the face of STEM with stormwater research

**DOI:** 10.1186/s40594-018-0099-2

**Published:** 2018-01-24

**Authors:** Mohamad Musavi, Wilhelm A. Friess, Cary James, Jennifer C. Isherwood

**Affiliations:** 1 College of Engineering, University of Maine, Orono, Maine USA; 2 Bangor High School, Bangor, Maine USA; 3 College of Education and Human Development, University of Maine, Orono, Maine USA

**Keywords:** High school research, Engineering and science practices, Stormwater, Citizen science, Mentor, Underrepresented in STEM, Experiential education, Community

## Abstract

**Background:**

The University of Maine Stormwater Management and Research Team (SMART) program began in 2014 with the goal of creating a diverse science-technology-engineering-math (STEM) pathway with community water research. The program engages female and underrepresented minority high school students in locally relevant STEM research. It focuses on creating educational experiences that are active and relevant to students that build confidence, connect knowledge and skills directly to solving problems in local communities, and support student cultural identities. The core tools of the SMART program are resources and relationships: university-designed or commercial water data collection equipment, data loggers and chemistry supplies, on-campus science and engineering training for teacher-mentors and students, and a community mentor network. The program supports an annual summer institute that trains both students and teacher-mentors and academic-year student research projects. SMART groups are formed at local schools or community centers. Activities revolve around engaging students in citizen-science to expand their understanding of the environment, developing community strategies to address the complex problem of stormwater pollution, and using the tools of science, engineering, and technology effectively. In addition, the program supports teachers and students in reaching out to local science and engineering professionals to form a mentor network for student research.

**Results:**

Over 3 years, 220 students and 25 teachers have been trained in the science and engineering of stormwater, having taken and recorded over 4000 local water measurements (i.e., temperature, conductivity, pH). In all cohorts to date, over 75% of student participants have self-identified as either female or a racial minority. Of approximately 125 currently college-eligible former and current SMART students, more than 41% have been accepted or are enrolled in a secondary STEM degree program. In pre- and post-program surveys, female and underrepresented minority students reported that SMART activities and their relationship with mentors have increased their awareness of how stormwater affects the community and increased their interest in pursuing a STEM career.

**Conclusion:**

With its focus on problem-solving at the community level, SMART supports students in active, local, and culturally relevant science and engineering experiences that contribute to building their confidence and affirming their decision to pursue post-secondary STEM careers.

## Introduction

The availability of clean water is a pressing and costly global challenge that affects life on both a global and local scale. Stormwater runoff is water from rain or melting snow that travels across impervious surfaces, such as pavement, instead of filtering into the ground. Runoff from agricultural land—and yards—can carry excess nutrients, such as nitrogen and phosphorus into streams, lakes, and groundwater supplies. These excess nutrients have the potential to degrade water quality. As it flows over the land surface, stormwater collects potential pollutants including sediment, nutrients from lawn fertilizers, bacteria from animal and human waste, pesticides from lawn and garden chemicals, metals from rooftops and roadways, and petroleum by-products from leaking vehicles that result in impairment of local water. Most individuals do not realize that stormwater drains directly into waterways and that the level of pollution in their local water bodies may be unhealthy. In addition to local watersheds, beach water quality generally declines following rain storms. Stormwater also impacts the function of combined sewer systems (CSSs), which collect rainwater runoff, domestic sewage, and industrial wastewater into one pipe. Under normal conditions, a CSS transports all of the wastewater it collects to a sewage treatment plant for treatment then discharges to a water body. The volume of wastewater can sometimes exceed the capacity of the CSS or treatment plant (e.g., during heavy rainfall events or snowmelt). When this occurs, untreated stormwater and wastewater discharge directly to nearby streams, rivers, and other water bodies. Combined sewer overflows contain untreated or partially treated human and industrial waste, toxic materials, and debris as well as stormwater.

The cost of stormwater management to municipalities is significant. To address this issue, some municipalities have begun imposing new stormwater fees on their residents. For example, about 21,000 property owners in the city of Portland, Maine are being charged a new fee that is based on the amount of hard or impervious surface on their property. Annual stormwater bills to property owners in Portland are now ranging from around $25 to more than $500. The new fee will generate revenue for the city’s $170 million in required upgrades to its stormwater system over the next 15 years, as mandated by the Environmental Protection Agency (Billings 2016).

Beyond the urban centers, polluted stormwater runoff is a concern in suburban and rural areas as it flows from roads, parking lots, farms, and yards into rivers and streams that directly feed into lakes and oceans. One of Maine’s Native American tribes, the Penobscot Nation have resided upon the Penobscot River waters and depended upon fish, plants, and wildlife from those waters for their physical, cultural, and spiritual sustenance for hundreds of years. Penobscot Nation students have participated in the SMART program, and their participation demonstrates the importance of providing a culturally meaningful STEM experience and establishing long-term relationships between mentors and students. SMART mentors have cultivated close relationships with Native American students by meeting students in their tribal community center and using a topic integral to their native culture and economy. Clam harvesting is an important source of livelihood for the Penobscot Nation community near Eastport, Maine, and the Native students in SMART have been researching the effects on stormwater pollution on the clam flats of the Sipayik River and Passamaquoddy Bay.

Whether implemented in urban or rural areas, the overall objective of SMART is to develop a meaningful and experiential STEM educational model that engages female and underrepresented minority (URM) students with a diverse community network. The representation of specific groups in STEM education and employment does not reflect their representation in the US population. Women, persons with disabilities, and three racial and ethnic groups—blacks, Hispanics, and American Indians or Alaska Natives—are underrepresented in STEM (NSF 2017).

Focusing efforts on females and minorities—but not excluding others—the specific objectives of SMART are to:

- Objective 1—Provide locally relevant and experiential educational opportunities for high school students and teachers,
- Objective 2—Support existing interest and increase confidence and enrollment in STEM fields,
- Objective 3—Stimulate interest in STEM through community engagement.

The SMART program began with a National Science Foundation (NSF) broadening participation award in 2013 for a program titled “Engineering Innovative Solutions to Stormwater Problems through Diverse Community Participation.” The program consists of two major components, a weeklong summer SMART institute and an academic year program. The first SMART training institute for students and teachers was held at The University of Maine (UMaine) in June 2014, and the first cohort of SMART students initiated school year activities during the academic year 2014–2015. The program now has active SMART groups at ten Maine high schools across the state and is currently undergoing a scale-up process to include eight additional states.

## Literature review

Addressing the problem of lack of diverse representation in STEM fields is intrinsically complex as its root causes are correspondingly complex. For females, causes for disengagement certainly have multiple origins and many are likely unique to each person’s circumstances. However, there are some commonalities cited in the literature:

- The culture of maleness in engineering and computer science (Bix 2004; Bystydzienski 2004; Evetts 1993; Sullivan 2007);
- Educational pathway issues (Blickenstaff 2005; Kohlstedt 2004);
- The absence of female scientists/engineers as role models (Morgan 1996; Neithardt 2007; Neimeier and González 2004).

Students from underrepresented groups, including female, report different experiences than majority students, even in the same STEM classes (Atman et al. 2010; National Academies of Sciences, Engineering, and Medicine 2016). These distinctions can lead to decreased confidence and an increased sense of work overload compared with males and majority students (Hoit and Ohland 1998). The issue is not ability in key STEM coursework but one of perceived ability. Research indicates that the male over female advantage in self-efficacy and beliefs about ability in math is stronger than ever; Ross et al. (2012) report that gender differences in beliefs prevail despite gender differences in achievement being near zero. Thus, in STEM fields, underrepresented minorities and females may be particularly vulnerable to disengagement (leaving a STEM field of study) due to beliefs about their ability to succeed in STEM, even when accounting for prior academic preparation (Litzler, Samuelson, and Lorah 2014; Moakler, Mikyong and Minsun 2014). However, belief in ability is strongly influenced by supportive mentors and believing that one’s ability in STEM can improve with learning and effort is related to positive motivational responses and performance outcomes (Grandy 1998; National Science Foundation 2005; Dai and Cromley 2014).

In response to these challenges, Changing the Conversation (National Academy of Sciences, National Academy of Engineering, and Institute of Medicine 2011) recommended that STEM fields such as engineering should communicate less about necessary engineering or science skills and more about the impact that students can have on the world. Invested, passionate mentors and role models demonstrate relationships between what students are learning and how it can change their community and the world (Ball 2011; Grossman and Parker 2012; Dyer-Barr 2013a, b). The crucial role of mentors especially for females and minorities is seen in other efforts. One example is a California Science Program in which participants identified the hands-on research and the mentor experience as the most valuable aspects of the program and reported increased science skills, increased confidence in science ability, and increased motivation and affirmation to pursue a science career (Salto et al. 2014). A sustained and long-term focus on student-centeredness, community building, and collaboration is an integral part of effectively implementing programs that target the recruitment and retention of URM students in STEM (Dyer-Barr 2013a, b; Salto et al. 2014).

To integrate diverse cultural backgrounds into STEM studies, students must first recognize and then negotiate and reconcile differences between their culturally based beliefs and those of mainstream science, which may not be obvious whatsoever to instructors and therefore be perceived as resistance or disengagement (Nelson-Barber and Estrin 1995). This obstacle is especially acute for Native American students, whose ways of knowing and views of the natural world are often very different from those in STEM classrooms (Aikenhead 1998; Aikenhead and Ogawa 2007). Native students may be alienated by STEM instruction that portrays scientific ways of knowing as disconnected from value and context, as such instruction conflicts with their cultural self-identity. Aikenhead (2001) boldly argues, “Only a small minority of students have worldviews and self-identities that align with the ways of knowing frequently conveyed in STEM classrooms.”

With a long-term collaboration of a higher education institution with local schools, and consistent support from community partners, the SMART program is working on connecting the classroom and the worldviews of students. The program includes a rapidly growing number of African American and Native American students. The higher education leaders and high school mentors are literally meeting minority and marginalized students “where they live” and actively demonstrating those crucial connections among water, culture, community, and science and engineering solutions.

## Program model

**Fig. 1:**
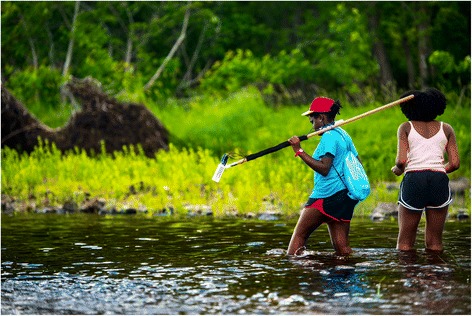
SMART student using a data logger on a tributary of the Penobscot River, Orono, ME

The SMART program model has been designed on the following principles to achieve its objectives: (a) be long-term (1 year or more); (b) provide mentorship; (c) include experiential learning, emphasizing engineering, and science practices; and (d) address a community-based environmental problem. The program starts with a summer institute on the UMaine campus for high school students and their teacher/mentors. The program continues with very active experiential learning throughout the following school year that culminates in a research product or presentation for each team of students. The summer institute trains students and teacher-mentors in using science and engineering skills and technology to research water quality in their local watershed. During the school year, students/teachers identify a waterbody in their local community with help from water district officials or scientists and participate in collecting data on a weekly basis (2–4 h), especially before and after a rain or storm (Fig. 1). Through this experience, students engage in numerous areas of STEM: engineering design, data acquisition, analysis and visualization, chemistry, environmental science, biology, and information technology. Teachers can integrate these areas in their existing courses, a specialized course, or after-school activities. Students also connect with a diversity of STEM professionals in water and engineering in government, private firms, and non-profits. The SMART program requires students to present their research publicly and engage in outreach to younger middle and elementary school students, as well as the local community, on the topics of stormwater science and engineering, to not only create awareness in the community, but also to encourage K-8 students to participate in STEM.

### Participant recruitment

For each of the first 3 years (2014–2016) of the SMART program, about 60–80 students and 7–10 teachers from ten Maine schools were selected for the summer institute. The program was advertised via statewide science and math teacher listservs and distribution of program fliers to schools and community organizations with high populations of URM students. Interested teachers contacted project staff at UMaine and discussed the time commitment and the process of recruiting students. Once a teacher committed to the program, s/he was asked to distribute program fliers widely at school. Students were guided to apply via an online application. Students must meet the basic qualifications of being a rising sophomore or junior, motivated to learn about science and engineering of water, having previous knowledge of chemistry and algebra (preferred), having a minimum GPA of 3.0, or a teacher’s recommendation. Students were required to obtain a teacher recommendation and answer two short essay questions regarding their interest in the program.

In 2017, the SMART program has piloted an expansion to eight additional states, including Alabama, California, Florida, Idaho, Mississippi, Missouri, New York, and North Carolina, with funds from the National Science Foundation’s INCLUDES (Inclusion across the Nation of Communities of Learners and Underrepresented Discoverers in Engineering and Science) program, aimed at collective efforts to broaden participation in STEM. This collaborative effort among Maine, New York, North Carolina, Florida, Alabama, Mississippi, Missouri, Idaho, and California brought together 20 teachers, 55 students, and 6 higher education faculty and personnel from all partner states to UMaine to learn about the SMART program and scale up the effort in their states. The 2017 SMART institute applied the lessons learned from the previous institutes and greatly expanded its teacher professional development component with the “cognitive apprenticeship through collaborative inquiry” model. In these sessions, and in practice with students throughout the week, teachers learned how to engage in collaborative inquiry with students on the science and engineering of stormwater.

### The summer institute

During each SMART summer institute, students and their teachers were trained at the University of Maine campus by faculty and graduate students. The program has been designed as a week-long residential program, which gives many students their first experience staying overnight in a college dorm and working in university laboratories.

During the institute work sessions, students and teachers directly monitor chemical, physical, and biological parameters of a local water body, the Penobscot River, taking measurements that include pH, conductivity, temperature, and flow rate. They are initially trained on the use of off-the-shelf Pasco sensors to monitor and establish a chemical profile of the river. This initial field study is conducted at the Orono wastewater treatment facility under the guidance of program staff. Facility staff gives institute participants a tour of the treatment plant to demonstrate how contaminated water is remediated. Participants are also trained on the use of kick nets to collect macro invertebrates and learn to identify various insects to establish species diversity indices of a river that assist in determining water quality.

Some water samples are taken off-site to the university analytical chemistry laboratory for determination of ortho and total phosphorous. Additional water samples are evaluated for total coliform and *Escherichia coli*, using an IDEXX Quanti-tray system, to determine the contamination level of the water body. To their surprise, students learn that the river has excessive levels of coliforms and levels greater than 15 ppb of phosphorus, placing it at risk of impairment. This leads to discussions about how they can remediate the problem and further think about how they might similarly assess impairment of local waterways in their communities. Students brainstorm about who they should contact in their communities to develop action plans both for the education of the general population and the development of possible remediation plans.

During their session at the river, students work in groups of two to three to collect environmental data. Data analysis is done later using data visualization and analysis tools including but not limited to Tuva Labs, Excel, and Google Earth. The collected data is pooled and populated into a single data file and then distributed to the students for the purpose of analysis. Once the data is obtained, students create presentations of the data and attempt to answer the question, “What is the story of the river?” They are directed to consider the importance of a longitudinal study that would allow for more data and a more accurate picture of a river’s response to stormwater events. Often, this leads students to look at stream data that are archived by local environmental groups such as the Penobscot River Keepers or the Volunteer Lake Monitoring Program.

**Fig. 2:**
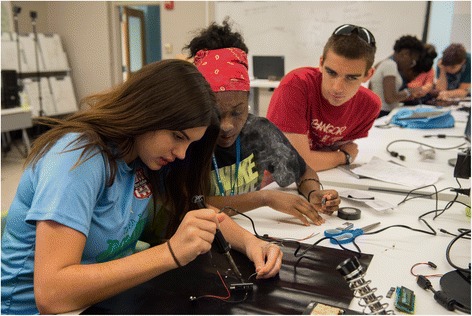
Students soldering circuits for their temperature sensors

The objective of the summer institute is to train students and teacher-mentors in using both science and engineering skills and technology to research water quality in their local watershed. Participants are trained in multiple aspects of engineering including electrical, chemical, computer, and environmental engineering. They are introduced to aspects of sensor design, looking specifically at the design of off-the-shelf sensors (Fig. 2).

**Fig. 3:**
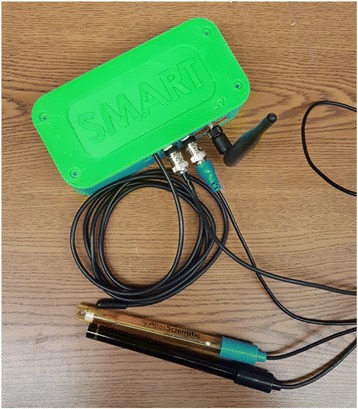
UMaine designed Wireless Stormwater Data Acquisition System

The engineering sessions at the institute culminate in having students build their own temperature and conductivity sensors. In the 2017 Institute, students used 3D printing to begin to design boxes (Fig. 3) that house the sensors, and 3D printed their own temperature sensors. These were field-tested at the institute and provided to each team for use in their year-long research.

### School year activities

xThe SMART teacher-mentors and their students are expected to meet weekly, committing to about 2–4 h per week that includes data collection and analysis, interaction with community partners, outreach in community, and providing a written summary at the end of the academic year describing their role in researching and managing stormwater. Student-collected water data includes parameters such as pH, conductivity, temperature, and flow rate as well as other in-lab information such as nitrate, chloride, ortho and total phosphorous, coliform, and other variables specific to water quality. The SMART groups develop action plans around the geographic-specific stormwater issues in their communities. Ideally, their action plans are developed with a community-based approach in conjunction with local stakeholders. Stakeholder group members are concerned with water quality or increasing diversity in STEM fields and include representatives from local universities and schools, departments of environmental protection, city planning offices, non-profit organizations advocating for the environment or diversity, engineering firms, and wastewater treatment facilities.

This broad community-based approach has resulted in students giving presentations at state and national science fairs, providing interactive demonstrations at youth water festivals, working with younger students in elementary schools, and presenting at town council meetings. Several SMART students have gone on to gain local and national recognition for their stormwater projects.

Students and teachers are supported during the year by the UMaine SMART project team with site visits, phone and email support, video-conferenced meetings, and online resources provided via the project website UMaine.edu/smart. These resources range from articles on stormwater to videos on technical lab procedures and to a Facebook page. In the 2017–2018 academic year, a monthly professional learning community for teachers is being led by the project team staff to maintain regular connection with the teacher-mentors and offer further development in the cognitive apprenticeship model of inquiry-based learning.

## Evaluation methodology

Four surveys were administered to students of any given cohort: pre-program in June, post-institute in July, mid-year in December, and post-program in May of the following year. While the post-institute and the mid-term surveys were designed and used to assess the participants’ perception of the SMART institute and their engagement in their engineering and science practices (these surveys are not presented here), the pre-program and post-program surveys were designed to assess the attainment of the project objectives. These surveys included questions about students’:

- Interest in STEM and non-STEM fields
- Level of knowledge in STEM fields
- Interest in higher education majors
- Interest in future careers
- Level of experience with hands-on tools and activities
- Level of confidence and comfort in taking STEM courses
- Level of interest in tackling real-world data-driven issues and finding a solution

The post-program survey also included questions on whether senior graduating students had applied to college and about their majors, as well as open-ended qualitative questions to express their opinion. Table 1 provides demographic data for students who participated in the SMART Institute. Race/ethnicity options in the survey questions were those listed in Table 1; the “others” category includes all other races including Asians. More than 90% of the participants completed the pre-program surveys. Table 2 gives the number of students that completed the post-program surveys. Participation in the surveys was voluntary. To improve the post-program survey completion, a small gift card was offered to those who completed their surveys in 2015 and 2016. As noted in Table 2, this increased the completion rate of the surveys to 71 and 72% during 2015 and 2016, respectively, as compared to 48% in 2014. These rates also include those students who dropped out of the program during the academic year. Among the most important factors for participating in the program were great opportunity to learn about STEM, new experiences, and topics that sounded interesting and fun.

**Table 1:** SMART student demographics

	Year (# of students)		
Students	2014 (61)	2015 (78)	2016 (81)
Male (%)	34	36	42
Female (%)	66	64	58
Caucasian (%)	43	59	54
Black (%)	26	10	15
Hispanic (%)	8	3	6
Native American (%)	16	18	10
Others (%)	5	6	15

**Table 2:** End-of-year student survey respondents

	Cohort 1 2014–2015	Cohort 2 2015–2016	Cohort 3 2016–2017
	*n* = 29	*n* = 57	*n* = 58
Female respondents (%)	72	60	62
URM respondents (%)	28	25	22
High school seniors (%)	66	37	62
High school seniors (female) (%)	45	23	41
High school seniors (URM) (%)	14	12	16

## Findings

The program was designed as a model to engage high school students in science and engineering-based experiential learning related to important local community issues, stormwater in this case. The results of this work are presented in the context of achieving the project objectives. Because the pre- and post-program surveys evolved over the three cohorts, it is difficult to quantitatively correlate longitudinal development from these differing surveys. However, longitudinal data have been presented as much as possible; otherwise, the results are for the specified cohorts. While the available information from different cohorts have been presented to provide quantitative data, the overall results are qualitatively consistent over the duration of the project.

### Experiential learning for high school students and teachers (objective 1)

From 2014–2016, students in the program conducted over 4000 water measurements at over 30 sites statewide and entered these data points (e.g., temperature, conductivity, pH) into a common online database. Students entered the program already attracted to STEM, with 70 to 58% of students indicating that they were *interested* or *very interested* in the various STEM areas—particularly biology, chemistry, environmental sciences, and engineering—in order of importance. Nevertheless, interest in these disciplines did not align with student knowledge of these disciplines, as reflected by many students indicating they knew nothing or almost nothing regarding many of these fields. The top five stormwater-related engineering and science practices, among 16 different project activities, with the greatest increases in participation rate are given in Table 3 for male and female students and in Table 4 for white and URM students. The top activities listed are related to collecting data via wireless sensors and probes and using them in real world applications. These activities surpass other important activities such as “designing solutions based on data,” “using computer modeling tools for data analysis,” and “recognizing different solutions based on different criteria.” Although there are slight differences in the ranking of the activities in Tables 3 and 4, the URM and female students demonstrated the highest change in participation. This result could be indicative of more interest in the selected activities and lack of prior experience in them. This result is also confirmed by the pre-program survey data that a majority of students looked forward to collecting data and learning new skills. Therefore, this information can inform educators on designing STEM activities that have the potential for attracting female and URM students.

**Table 3:** The top five activities with the greatest increase in average participation rates for male and female students (2014–2015 cohort, as given in Table 2)

Male		Female	
Activity	Change	Activity	Change
	Pre–post (%)		Pre–post (%)
Using wireless sensors for data	0–88	Collecting water data via probe	35–88
Building a wireless sensor network	13–88	Using wireless sensors for data	13–65
Collecting data via sampling	63–100	Building a wireless sensor network	6–53
Using data to solve world issues	50–75	Collecting water data via sampling	59–100
Collecting water data via probe	63–88	Using sensor technology for data	47–88

**Table 4:** The top five activities with the greatest increase in average participation rates for white and URM students (2014–2015 cohort, as given in Table 2)

White		URM	
Activity	Change	Activity	Change
	Pre–post (%)		Pre–post (%)
Using wireless sensors for data	13–69	Using wireless sensors for data	0–75
Collecting data via sampling	56–100	Building a wireless sensor network	0–75
Building a wireless sensor network	12–50	Using sensor technology for data	25–88
Collecting water data via probe	50–88	Collecting non-water data via probe	38–88
Using sensor technology for data	56–88	Collecting water data via probe	38–88

Students in the 2015 cohort reported that numerous key components of the summer institute made them *somewhat* or *to a great extent* more likely to apply to a STEM program in college. These included participating in real-world, community-based research opportunities (83.1%), and then using these experiences to research real issues facing their community (93.7%), and helping to solve these local problems (95.3%). These data highlight the importance students place on finding solutions and feeling empowered to address these problems by connecting with their community (objective 1). SMART provides students with the abovementioned opportunities across the academic year.

### Improve enrollment of female and minorities in STEM education (objective 2)

**Fig. 4:**
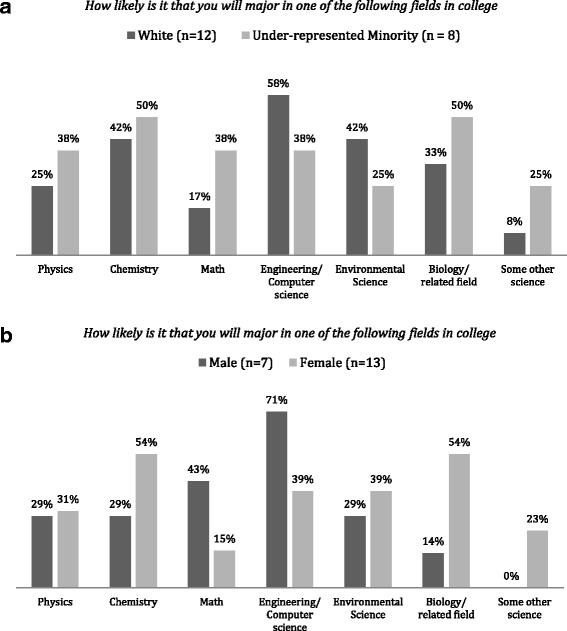
**a**, **b** The result of a survey about the likelihood of majoring in STEM majors administered to participating 2015–2016 cohort 1 year after the SMART institute

Project data also suggests that the program design and experiences are effective in promoting interest in STEM education and careers among participants (objective 2). More than 50% of all respondents said the summer institute experiences encouraged them “*To a Greater Extent*” to apply for college STEM program. For female students, hands-on data collection at streams, also given in Table 3, and using STEM skills to solve real problems were the most important factors for being attracted to STEM fields. For URM students, these factors were working with wireless sensors (Fig. 4), experience of staying in a college campus, and STEM skills to solve real problems. Learning about possible jobs in STEM was also an important factor in choosing a STEM-related major. Figure 4 shows the distribution of students and their planned fields of study. While male students show more interest in engineering (57%), female students are more interested in biology and chemistry. Similar comparison exists between white and URM students.

Ultimately, the goal of SMART is to improve enrollment, especially among female and minority students, in post-secondary STEM fields. Of approximately 125 former and current SMART students who are currently college-eligible (they have graduated or are ready to graduate from high school), more than 41% (51) have been accepted or are enrolled in a secondary STEM degree program and 84% of these STEM-enrolled students are either female or URM. Note that while this percentage is close to the percentage of female and URM student participants in the program, they typically enroll in STEM secondary education at a lower rate than their white male peers. From the 2014 cohort alone, 19 of the 20 graduating high school students who completed post-program surveys reported that they are continuing into post-secondary education. While this far exceeds the statewide average of 62% of seniors attending college in the following year, making a quantitative comparison to the general population remains difficult. This is due to the fact that the recruiting mechanism of SMART specifically targets academically prepared students, and no general data relating secondary academic performance with post-secondary STEM attendance is available for Maine schools. From these 19 students, 7 are in biology, 4 in engineering, and 4 in environmental sciences with more male students in engineering and more female and URM students in biology and environmental sciences.

The program has demonstrated that it can promote and sustain interest in STEM education and careers specifically for females and minorities. Table 5 displays the change in level of interest toward a STEM degree or career for the different student demographic groups.

**Table 5:** Change in level of interest in a STEM college or career after completing SMART 2015 Cohort

How has your level of interest in choosing a STEM major/career changed after participation in the SMART program	Male (*n =* 20)		Female (*n =* 37)		White (*n =* 39)		URM (*n =* 18)		Students responded (*n =* 57)	
	*n*	Pct. (%)	*n*	Pct. (%)	*n*	Pct. (%)	*n*	Pct. (%)	*n*	Pct. (%)
I am less interested	0	0.0	1	2.7	1	2.6	0	0.0	1	1.8
My interest has not changed	9	45.0	20	54.1	18	46.2	11	61.1	29	50.9
I am more interested	11	55.0	16	43.2	20	51.3	7	38.9	27	47.4

**Fig. 5:**
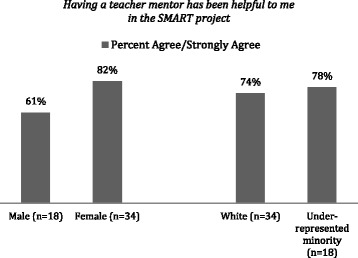
Survey administered to 2015–2016 cohort 1 year after the SMART institute to assess perceived importance of mentors

The SMART program model also stresses the importance of long-term relationships between mentors and students. This is best evidenced by the close relationships SMART mentors have established with Native American students by using a topic integral to their native culture and economy. In all cases, the teacher-mentors promoted the interest in the students and maintained and channeled this interest throughout the yearlong extracurricular activities. Figure 5 illustrates the perceived importance of the teacher-mentors in student individual success in the program; in particular, the female and URM students were appreciative of these mentors and the role they played in their success.

**Fig. 6:**
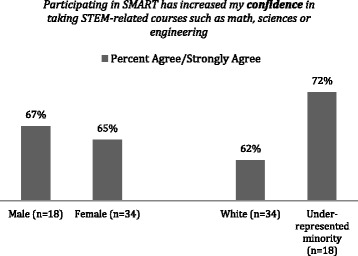
Survey administered to 2015–2016 cohort 1 year after the SMART institute, showing increased perceived confidence in STEM related abilities

The combination of mentorship and addressing a global problem in the local community has shown to strengthen the students’ confidence to succeed in STEM. This effect is particularly noticeable in the URM students as Fig. 6 shows.

### STEM education through community engagement (objective 3)

**Fig. 7:**
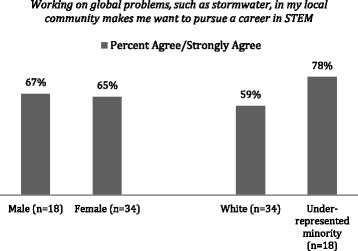
Survey results for 2015–2016 participating HS seniors 1 year after the SMART institute illustrating the perceived importance of community relevance of the experience

A key component in stimulating interest in the program and consequently a STEM career is the combination of a global problem in a community environment. Collected summative data highlight the importance students place on finding solutions and feeling empowered to address these problems by connecting with their community (Fig. 7).

Without projects such as SMART, few students have opportunities to work on real world problems in their communities. Prior to participation, nearly half of students (48.6%) indicated that they had never used data to help solve a local environmental problem, and three fourths (74.3%) indicated that they had never worked with environmental officials. SMART provides students with many hands-on activities to work on the local environmental issue of stormwater across a full academic year and beyond, if desired.

Local water districts have supported the SMART program by providing technical expertise, presentations, and identifying appropriate watershed locations for water sampling and installation of wireless sensors. Water districts are obligated to perform community outreach and provide water quality data to the Environmental Protection Agency (EPA) on a regular basis. Therefore, the districts gain from the program by engaging in mutually beneficial activities with a local SMART group, and also by having access to a trained workforce for internships or future permanent jobs, even beginning at the high school level.

The combination of educating Maine students in STEM while also addressing locally relevant issues provides a powerful incentive for the engagement of community organizations. Organizations that have supported the UMaine SMART program include IDEXX Corporation, Emera Maine (an electric power utility), non-profit Maine Community foundation, and Bangor Savings Bank. IDEXX is a leader in diagnostics and IT solutions for animal health and water/milk quality and to date has supported SMART by providing thousands of tests and measurement devices to be used by students for measuring *E. coli* and total coliforms in water. These organizations have contributed funds and equipment in the last 4 years to augment the NSF award. With their support, the program was able to increase the participation of students from 60 students/year to 80 students/year.

## Conclusions

The SMART program began in 2014 to increase the participation of females and minorities in STEM at the high school level, with the long-term goal of increasing student enrollment in post-secondary STEM programs as well as participation in the workforce.

To date, SMART has trained 220 students and 25 teachers to mentor and guide students in their activities and education. In the 2014–2016 cohorts, 75–84% of SMART student participants have self-identified as either female or a racial minority. Of approximately 125 currently college-eligible former and current SMART students, more than 41% (51) have been accepted or are enrolled in a secondary STEM degree program; 84% of these STEM-enrolled students are either female or URM.

The combination of mentorship and addressing a global problem in the local community has been shown to strengthen the students’ confidence to succeed in STEM, with 72% of participating URM students reporting increased confidence in taking STEM courses. Female students (82%) and URM students (78%) strongly agree that having a teacher-mentor—the strongest factor among all other features of the SMART program—is beneficial in their motivation toward STEM.

The program has provided advanced instrumentation to the schools and motivated the engagement of a range of community and industrial organizations in support of SMART, allowing the program to increase the participation of students in the program from 60 students/year to 80 students/year. SMART student groups are connecting with their community water and engineering professionals and younger students to interact and learn more about the issue of stormwater contamination to Maine’s local water bodies. SMART students have presented to their local boards of education, stenciled storm drains, regularly participated in elementary STEM clubs, and led hands-on activities at Maine’s Children’s Water Festival—which annually brings together more than 600 students and teachers around water. In the last 3 years, SMART students have entered in several science competitions in Maine and nationally, presenting their stormwater research; several have been awarded first and second place in the Intel Science Talent Search and Stockholm Junior Water Prize.

The SMART program to date has now evolved into a state-wide program, and current development and growth activities are aligned with the NSF INCLUDES expansion to a multi-state collaborative. This scale-up is addressing some of the challenges encountered throughout the implementation of the SMART program during the past years. These include the success of a local SMART group hinging on a single motivated teacher carrying the weight of the program at his or her school. The scale-up is addressing these concerns through social networking research and a systematic expansion of the community-based support mechanisms to ensure sustainability beyond the single-person efforts. Further, the collaborative is developing an integrated assessment process for both the broadening participation outcomes and the scale-up process, to improve the availability of longitudinal data and allow multi-state longitudinal data collection and evaluation. This assessment method will extend to post-secondary to document the longitudinal effectiveness of the program.

The SMART program has also posed challenges that are being addressed in the current program. One of these challenges is the attrition of the SMART students, often attributed to the lack of available after-school time for SMART activities due to sports, other extracurricular activities, or part-time jobs. This could be remedied in some schools by including SMART activities as part of existing courses in the high school curriculum or creating a new course. On the technical aspects of SMART activities, the greatest challenge was the loss of wireless sensor data acquisition systems, due to vandalism, when left unattended in a watershed over a long period of time over. This was resolved by employing secure locations, controlled by water districts, or collecting intermittent data before or after a rainstorm. Furthermore, in places where WiFi (the communication protocol for our wireless sensor data acquisition system) access was limited, real-time data could not be collected. Finally, lack of teachers’ experience with teaching of engineering and hands-on science and mentoring students could be detrimental to the success of the program. This has led the project team to plan for teacher-only professional development in collaborative inquiry through cognitive apprenticeship, as a part of the summer institute.

Overall, the SMART program has demonstrated that local, long-term, hands-on environmental research in a guided mentorship program can support and sustain the interest of female and URM students, increasing their confidence and enrollment in STEM fields.

## Abbreviations

NSF: National Science Foundation
SMART: Stormwater Management and Research Team
STEM: Science, technology, engineering, and math
URM: Underrepresented minority
